# Causes and consequences of stress generation: Longitudinal associations of negative events, aggressive behaviors, rumination, and depressive symptoms

**DOI:** 10.1007/s12144-022-02859-9

**Published:** 2022-02-23

**Authors:** Akira Hasegawa, Shin-ichi Oura, Tetsuya Yamamoto, Yoshihiko Kunisato, Yuko Matsuda, Masaki Adachi

**Affiliations:** 1 Faculty of Human Relations, Tokai Gakuin University, 5-68 Naka-kirino, Kakamigahara City, Gifu, 504-8511 Japan; 2 Graduate School of Technology, Industrial and Social Sciences, Tokushima University, 1-1, Minamijosanjima-cho, Tokushima, 770-8502 Japan; 3 Department of Psychology, School of Human Sciences, Senshu University, 2-1-1, Higashimita, Tama-ku, Kawasaki-shi, Kanagawa 214-8580 Japan; 4 Graduate Schools of Health Sciences, Hirosaki University, 66-1, Hon-cho, Hirosaki-shi, Aomori, 036-8564 Japan

**Keywords:** Stress generation, Negative event, Aggression, Rumination, Depression

## Abstract

The present study examined the causes and consequences of stress generation in university students in Japan. A two-wave longitudinal study with an 8- or 9-week interval was conducted in the fall of 2020. Undergraduate and graduate students at four universities in Japan (*N* = 201) completed self-report measures assessing experiences of negative interpersonal dependent events, negative non-interpersonal events, and negative independent events at two times. At the same time, they also responded to measures of aggressive behaviors, trait rumination, and depressive symptoms. Path analyses revealed that baseline aggressive behaviors were positively associated with an increase in subsequent negative interpersonal dependent events, even after controlling for the influences of negative interpersonal dependent events, rumination, and depressive symptoms at baseline. However, aggressive behaviors were not significantly associated with subsequent negative non-interpersonal dependent events or negative independent events. These findings suggest that aggressive behaviors may have been a factor leading to interpersonal stress generation. Furthermore, all categories of negative event experiences predicted an increase in subsequent depressive symptoms, but not subsequent rumination, and rumination was not significantly associated with subsequent depressive symptoms. This research extends previous studies on the causes and consequences of stress generation conducted in the US by using specific measures of aggressive behaviors and including a non-restricted sample of university students in Japan.

**Supplementary Information:**

The online version contains supplementary material available at 10.1007/s12144-022-02859-9.

Early studies on stress and depression have mainly focused on the effect of negative life events on the risk of developing depressive symptoms and depressive disorders (see Hammen, 2005; Monroe et al., 2014, for reviews). On the other hand, the stress generation hypothesis (Hammen, 1991) assumes that depression-prone individuals are active agents in the creation of negative events. Stress generation research has emphasized the distinction between dependent events resulting from people’s characteristics and behaviors, such as interpersonal conflicts, and independent events occurring outside the individual's control, such as the death of a relative or friend. Previous studies have shown that individuals with depressive disorders or subclinical depressive symptoms, in contrast to their non-depressed counterparts, experience frequent negative dependent events, particularly in the interpersonal domain. However, this effect is not observed for negative independent events (see Liu & Alloy, 2010, for a review). Previous studies have also suggested that negative life events generated by the self can predict a future increase in depressive symptoms (Belmans et al., 2019; Flynn et al., 2010; Flynn & Rudolph, 2011; Hankin et al., 2010; Snyder & Hankin, 2016) as well as the recurrence of major depression (Bos et al., 2007). Therefore, the stress generation hypothesis holds that person-environment interactions exacerbate depression.

Previous studies have also examined cognitive and behavioral factors leading to stress generation. Aggressive behaviors are considered to be one such factor. Because aggressive behaviors are acts by which others are physically or psychologically harmed, it is reasonable to assume that these behaviors may be a cause of interpersonal stress generation. A few longitudinal studies conducted in the US have supported this assumption. For example, a study with adolescent girls by Stroud et al. (2015) found that scores on the aggression subscale of the Multidimensional Personality Questionnaire-Brief Form (Patrick et al., 2002) were associated with an increase in acute interpersonal dependent stressors during the subsequent one year, even after controlling for baseline depressive symptoms and rumination. Similarly, a study of university students with bipolar spectrum disorders and others with no Axis I psychiatric disorder suggested that scores on the physical aggression subscale, as well as scores on the hostility and anger subscales of the Aggression Questionnaire (Buss & Perry, 1992), were positively associated with subsequent anger-evoking events (Molz et al., 2013).

Although these previous results suggest that aggressive behaviors may be a factor in stress generation, very few studies have examined this possibility. In addition, previous studies had been conducted only in the US. Therefore, it remains unclear whether these results apply to non-Western populations. Markus and Kitayama (1991) suggested that Western and Asian people have strikingly different self-construals. They theorized that the former sees the self as separated from others, and the latter sees the self as interdependent with others in significant social units. Researchers have assumed that Westerns' self-esteem is dependent on the ability to express the self and validate internal attributes, whereas that of Asians is dependent on the ability to maintain harmony within the social context (see also Kitayama et al., 2009; Park et al., 2016). Therefore, the functions of aggressive behaviors within the social context might differ between Westerns and Asians.

The study by Stroud et al. (2015) also had specific limitations in that the study included only female adolescent participants and did not use a specific measure of aggressive behaviors. Furthermore, Molz et al. (2013) included only university students with specific characteristics (i.e., students with bipolar disorders and those without any Axis I disorders) in the study sample. Therefore, it is uncertain whether their findings ate applicable to university students in general. Considering these limitations, the present study attempted to obtain further evidence of the longitudinal relationship between aggressive behaviors and interpersonal stress generation in university students in Japan. This study also addressed the following issues.

This study examined whether aggressive behaviors predict subsequent negative interpersonal dependent events even after controlling for the influence of depressive symptoms and rumination. As described above, depressive symptoms have been a consistent predictor of increased negative dependent events (Liu & Alloy, 2010). Moreover, rumination might be a factor in stress generation. Rumination is defined in the response styles theory as “behaviors and thoughts that focus one’s attention on one’s depressive symptoms and on the implications of these symptoms” (Nolen-Hoeksema, 1991, p. 569), which is demonstrated to be a robust predictor of depression (see Nolen-Hoeksema et al., 2008, for a review). Previous studies have suggested that rumination might lead to stress generation, particularly in the interpersonal domain (Flynn et al., 2010; McLaughlin & Nolen-Hoeksema, 2012; Shapero et al., 2013a; Stroud et al., 2015), although negative results have also been obtained (Hamilton et al., 2013, 2017; Shapero et al., 2013b). A longitudinal study has reported positive associations between aggressive behaviors, depressive symptoms, and rumination (McLaughlin et al., 2014). Therefore, it is essential to examine whether the association between aggressive behaviors and subsequent negative interpersonal dependent events can be found without confounding depressive symptoms and rumination. Stroud et al. (2015) found an association between baseline aggression and subsequent acute interpersonal dependent stressors after controlling for the influences of baseline acute interpersonal dependent stressors as well as depressive symptoms and rumination. In the present study, we examined whether similar partial associations can be obtained.

In addition, this study examined associations of negative interpersonal dependent events, negative non-interpersonal dependent events, and negative independent events with subsequent depressive symptoms and rumination. Although previous research has suggested that concurrent correlations with depressive symptoms are significantly stronger for negative dependent events than for negative independent events (Hasegawa et al., 2021b), the reason for this remains unclear. This result might indicate that depressive symptoms predict negative dependent events but not negative independent events (Liu & Alloy, 2010). It is also plausible that negative dependent events have a greater effect on depressive symptoms than do negative independent events, because attributing negative events to internal causes is associated with depressive symptoms (Huang, 2015). A longitudinal study should reveal which of these explanations is more adequate.

Furthermore, the goal progress theory of rumination assumes that negative events can prevent a person from attaining goals, which leads to rumination about goals that are yet to be attained (Martin et al., 2004). In fact, previous studies have demonstrated that frequent exposure to negative events is prospectively associated with increased rumination (Michl et al., 2013). Moreover, among negative interpersonal dependent events, negative non-interpersonal dependent events, and negative independent events, only negative interpersonal dependent events have been longitudinally associated with an increase in rumination after controlling for the influences of other categories of events (Hamilton et al., 2015). An explanation may lie in the psychobiological theory of depression (Slavich et al., 2010), which proposes that because humans have a fundamental drive to maintain positive social status, social values, and social regard, social rejections that threaten these needs may elicit negative self-referential cognitions concerning social worth and self-esteem (i.e., rumination). Although these findings are important in explaining the causes of rumination, few studies have examined the association between negative events and rumination assessed at a later time point.

The present study also examined the relationship between rumination and subsequent depressive symptoms after controlling for the influences of aggressive behaviors and each category of negative events assessed in stress generation research. Although many longitudinal studies have shown that trait rumination is associated with an increase in depressive symptoms (Nolen-Hoeksema et al., 2008), to our knowledge, no previous study has examined this partial association. This investigation should provide additional evidence about the causes and consequences of stress generation and rumination.

In summary, a short-term longitudinal study was conducted to examine the longitudinal relationships between aggressive behaviors, three categories of negative events in the stress generation research context, rumination, and depressive symptoms in university students in Japan. This study examined four hypotheses. Firstly, we hypothesized that aggressive behaviors at baseline would be positively associated with subsequent experiences of negative interpersonal dependent events, even after controlling for the influence of baseline negative interpersonal dependent events as well as depressive symptoms and rumination consistently with previous studies showing the effect of aggressive behaviors on interpersonal stress generation (Molz et al., 2013; Stroud et al., 2015). We also predicted that aggressive behaviors would not be significantly associated with subsequent experiences of negative non-interpersonal dependent events and negative independent events (Hypothesis 1). In addition, because many previous studies have demonstrated that the experience of negative events could increase the risk of developing depressive symptoms and depressive disorders (Hammen, 2005; Monroe et al., 2014), we predicted that all categories of negative events would be associated with an increase in depressive symptoms (Hypothesis 2). Furthermore, based on the goal progress theory of rumination (Martin et al., 2004) and the findings of Michl et al. (2013), we predicted that all categories of negative events would be associated with subsequent rumination (Hypothesis 3). We also explored whether the associations with depressive symptoms and rumination would differ for each category of negative events. Finally, in line with previous findings indicating that rumination is a robust predictor of depression (Nolen-Hoeksema et al., 2008), we hypothesized that rumination would be associated with increased depressive symptoms (Hypothesis 4).

## Method

### Participants

Participants were recruited in classes at Hirosaki University, Senshu University, Tokai Gakuin University, and Tokushima University in Japan. Undergraduate and graduate students (*n* = 437) in these universities responded to a packet of questionnaires during September and October 2020 (Time 1). Most participants answered the questionnaires again 8 weeks later (Time 2) in the same classroom in which they had been recruited at Time 1. However, 25 participants answered the questionnaires 9 weeks after Time 1 because their class could not be held 8 weeks later due to the spread of COVID-19. In addition, 36 participants at Tokushima University and 119 undergraduate students at Tokai Gakuin University who participated at Time 1 could not respond to the questionnaires at Time 2 because their classes could not be conducted face-to-face on the day originally scheduled as Time 2, for the same reason. Although these classes were held online at Time 2, online surveys could not be conducted because the Japanese version of the Beck Depression Inventory-Second Edition, one of the scales used in this study, was prohibited from being used online by Nihon Bunka Kagakusha Co., Ltd. In total, 259 students answered the questionnaire 8 or 9 weeks after Time 1.^1^

The data of participants who did not participate at both time points and those with missing data on any questionnaire at either time were excluded from the analyses. The final sample comprised 201 students (84 men, 117 women). The mean age of the final sample was 20.20 (*SD* = 2.83, age range 18–51 years) at Time 1. All participants were Japanese, except for 3 participants (one Chinese, one Korean, and one Thai).

### Measures

#### Aggression Scale (Isobe & Hishinuma, 2007)

This scale is a measure of aggressive behaviors, which was developed in a study of Japanese university students. This study used only the items of the overt aggression subscale for assessing aggressive behaviors. This subscale is composed of 12 items having face validity assessing physical and verbal aggressive behaviors, including “sometimes I was violent unintentionally” and “sometimes I was sarcastic and said bad things to others’ faces.” This subscale showed good internal consistency (Isobe & Hishinuma, 2007). In addition, the overt aggression subscale showed a concurrent association with increased negative interpersonal events but not negative achievement events (Hasegawa et al., 2021a), indicating acceptable construct validity. Each item was rated on a five-point rating scale using the anchors 1 (*not at all true of me*) and 5 (*extremely true of me*). The overt aggression subscale demonstrated sufficient internal consistencies at Time 1 and 2 (*α*s = .83 and .84, respectively).

#### Negative Independent/Dependent Events Scale (Hasegawa et al., 2021b)

This scale is a self-report measure assessing experiences of negative events in Japanese university students that was designed to test the stress generation hypothesis. The Negative Independent/Dependent Events Scale contains 25 items on the negative interpersonal dependent events subscale (e.g., “I had a terrible relationship with my friend”), 14 items on the negative non-interpersonal dependent events subscale (e.g., “My report was badly graded”), and 20 items on the negative independent events subscale (e.g., “I faced a disaster such as heavy rain or snow”). This scale showed acceptable construct validity in a study of a sample of Japanese university students (Hasegawa et al., 2021b). Participants were asked to respond to the extent to which they had experienced each event in the last 8 weeks using a rating scale ranging from 1 (*never*) to 4 (*often*). Alpha coefficients at Time 1 and 2 were .91 and .92 for the negative interpersonal dependent events subscale, .82 and .83 for the negative non-interpersonal dependent events subscale, and .80 and .83 for the negative independent events subscale.

#### Ruminative Responses Scale (RRS; Nolen-Hoeksema & Morrow, 1991)

The RRS is a scale of trait rumination. This scale includes 22 items, each rated on a 4-point rating scale anchored at 1 (*almost never*) and 4 (*almost always*). Five items in the RRS assess brooding (e.g., “Think ‘Why do I have problems other people don’t have?’”), five items assess reflection (e.g., “Analyze recent events to try to understand why you are depressed”), and 12 depression-related items (e.g., “Think about how sad you feel”; Treynor et al., 2003). We calculated brooding and reflection subscale scores and the total RRS score. Adequate psychometric properties of the RRS, including good internal consistency and construct validity and moderate test-retest reliability of the total and subscale scores, have been reported (Schoofs et al., 2010; Treynor et al., 2003). The Japanese translation of the RRS was used, which has the same psychometric properties as the original version (Hasegawa, 2013). Alpha coefficients at Time 1 and 2 were .95 and .95 for the overall RRS, .86 and .85 for the brooding subscale, and .76 and .79 for the reflection subscale in our sample.

#### Beck Depression Inventory-Second Edition (BDI-II; Beck et al., 1996)

The BDI-II is a well-validated questionnaire assessing the severity of depressive symptoms experienced in the past two weeks. Participants respond to 21 items using a 0–3 scale, with higher scores indicating more severe depression. The Japanese translation of the BDI-II by Kojima and Furukawa (2003) was used. The BDI-II showed good reliability and validity as the original version (Beck et al., 1996) and the Japanese version (Kojima & Furukawa, 2003). The BDI-II showed excellent internal consistency at Time 1 and 2 (both *α*s = .92) in our sample.

### Procedure

Students were given a description of the study prior to participation. Only the students who agreed to take part in the study responded to the questionnaires. Participants answered all scales described above at Time 1 and 2. We set 8 weeks as the interval between Time 1 and 2 because we considered that this interval would be required to detect the effect of the aggressive behaviors on subsequent negative interpersonal dependent events.^2^ In addition, this interval facilitated conducting two surveys with an 8-week interval within the same semester.

Participants wrote their birthdays and the last four digits of their mobile phone numbers on spaces provided in the questionnaire packet, which were used to match the data from the surveys at the two times. The Ethics Committee of Tokai Gakuin University approved the study.

### Statistical Analysis

Data were analyzed by allowing for missing data. Descriptive statistics and *t*-tests were conducted using SPSS ver. 23 (IBM Corporation), and all other analyses were conducted using Mplus 8.3 (Muthén & Muthén, 1998–2017). Simple correlations among all variables were calculated. Path analysis with maximum likelihood estimation method was conducted to examine whether aggressive behaviors, each category of negative events, rumination, and depressive symptoms at Time 1 were predictive of each variable at Time 2. We examined separate models using one of the subscales for negative interpersonal dependent events, negative non-interpersonal dependent events, and negative independent events. Although we used total RRS scores as the main variable of rumination, we conducted supplementary path analyses using either brooding or reflection subscales. In each model, correlations between all variables at Time 1 and those between error variables of all study variables at Time 2 were assumed.

Little’s MCAR test (1988) yielded a non-significant chi-square value (*χ*^2^ (201) = 233.58, *p* = .057), indicating that values were missing at random. Missing data were handled with multiple imputations using Bayesian analysis when conducting correlation analyses and path analyses. Imputation was conducted using age, gender, and all items of study variables, and 20 data sets were generated and used for analyses. In other analyses, missing data were handled with the pairwise method.

Because some studies have suggested that influences of psychological predictors on stress generation are stronger among females than males (Liu & Alloy, 2010), we initially planned to collect data from at least 150 male and 150 female participants in order to conduct a multiple group path analysis with gender of each participant as a grouping variable. However, because of the spread of COVID-19 in Japan, a sample of sufficient size could not be collected, and we were unable to compare longitudinal associations of each variable between male and female groups.

## Results

Descriptive statistics of the final sample are shown in Table 1. Paired *t*-tests were performed using the data from participants who completed each subscale at both Time 1 and 2 without missing values. No variables had significantly different scores between Time 1 and Time 2 (*t*s < 1.38, *p*s > .169, *d*s < 0.04).^3^ Table 2 shows the correlations between each of the study variables.

**Table 1:** Descriptive statistics for each measure

	*n*	*M*	*SD*	Range	Skewness	Kurtosis
Time 1						
Aggressive behaviors	200	23.80	7.75	12 – 46	0.89	0.15
Negative interpersonal dependent events	197	37.20	10.44	25 – 71	0.98	0.32
Negative non-interpersonal dependent events	198	23.89	6.57	14 – 48	0.79	0.31
Negative independent events	199	29.40	6.76	20 – 59	1.07	1.40
Brooding	200	10.69	4.15	5 – 20	0.39	–0.74
Reflection	201	8.70	3.29	5 – 18	0.73	–0.27
Rumination total	199	43.85	14.76	22 – 84	0.49	–0.35
Depressive symptoms	196	13.36	10.16	0 – 56	1.18	1.57
Time 2						
Aggressive behaviors	200	23.32	7.87	12 – 47	1.13	0.79
Negative interpersonal dependent events	199	37.09	10.69	25 – 90	1.36	2.74
Negative non-interpersonal dependent events	200	23.43	6.46	14 – 49	0.91	0.84
Negative independent events	201	28.85	7.16	20 – 59	1.56	2.94
Brooding	199	10.75	4.09	5 – 20	0.36	–0.71
Reflection	201	8.72	3.47	5 – 20	0.95	0.29
Rumination total	199	43.89	14.99	22 – 82	0.49	–0.54
Depressive symptoms	198	12.92	9.98	0 – 49	0.99	0.83

**Table 2:** Correlations between variables

	1	2	3	4	5	6	7	8	9	10	11	12	13	14	15
Time 1															
1. Aggressive behaviors															
2. Negative interpersonal dependent events	.32														
3. Negative non-interpersonal dependent events	.27	.66													
4. Negative independent events	.32	.69	.69												
5. Brooding	.15	.47	.52	.43											
6. Reflection	.22	.44	.43	.39	.56										
7. Rumination total	.19	.50	.54	.46	.90	.77									
8. Depressive symptoms	.15	.47	.50	.37	.62	.39	.66								
Time 2															
9. Aggressive behaviors	.75	.27	.25	.29	.09	.15	.14	.15							
10. Negative interpersonal dependent events	.33	.70	.52	.48	.31	.36	.35	.34	.38						
11. Negative non-interpersonal dependent events	.18	.52	.68	.50	.35	.32	.37	.40	.26	.61					
12. Negative independent events	.23	.49	.48	.63	.26	.24	.27	.30	.32	.66	.67				
13. Brooding	.10	.40	.38	.32	.63	.52	.65	.56	.16	.46	.39	.32			
14. Reflection	.12	.33	.30	.29	.33	.68	.50	.34	.15	.41	.35	.31	.60		
15. Rumination total	.11	.41	.42	.36	.58	.63	.70	.60	.17	.49	.44	.37	.92	.79	
16. Depressive symptoms	.13	.49	.51	.40	.51	.34	.54	.84	.17	.46	.51	.39	.61	.39	.66

Absolute correlations of .14 or higher are significant at *p* < .05

**Fig. 1:**
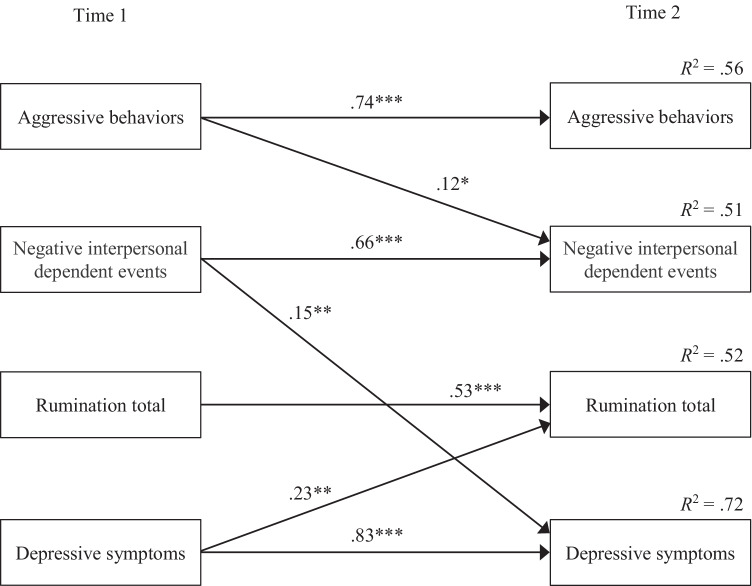
The results of the path analysis using negative interpersonal dependent events as a stressor measure and total RRS score as a rumination measure (*N* = 201). Only paths with significant standardized regression coefficients at 5% level are shown. Non-significant paths, error variables, and covariances are omitted. *p < .05, **p < .01, ***p < .001.

Table 3 shows the results of the path analyses using total RRS score as an index of rumination. There was no test of overall model fit because all models were saturated (i.e., zero degrees of freedom). The model using negative interpersonal dependent events as a measure of negative events indicates that aggressive behaviors at Time 1 were significantly related to increased negative event experiences at Time 2. On the other hand, baseline aggressive behaviors had nonsignificant associations with subsequent negative non-interpersonal dependent events and negative independent events. All categories of negative events at Time 1 were associated with an increase in depressive symptoms at Time 2. Baseline rumination was not significantly associated with subsequent depressive symptoms. In all models, depressive symptoms at Time 1 were predictive of an increase in rumination at Time 2, but all categories of negative events were not predictive of later rumination. Aggressive behaviors at Time 2 were predicted by aggressive behaviors at Time 1 but by no other variables. The results of the path model using negative interpersonal dependent events as a stressor measure are depicted in Fig. 1.

**Table 3:** Standardized estimates for each path analysis model

	Negative interpersonal dependent events as a stressor measure			
Independent variables	Aggressive behaviors T2	Negative interpersonal dependent events T2	Rumination total T2	Depressive symptoms T2
Aggressive behaviors T1	.74 ***	.12 *	–.04	–.03
	[.67, .81]	[.01, .22]	[–.14, .06]	[–.10, .05]
Negative interpersonal dependent events T1	.04	.66 ***	.05	.15 **
	[–.07, .15]	[.56, .76]	[–.06, .17]	[.06, .24]
Rumination total T1	–.06	–.01	.53 ***	–.08
	[–.19, .06]	[–.15, .13]	[.41, .65]	[–.18, .03]
Depressive symptoms T1	.07	.02	.23 **	.83 ***
	[–.06, .19]	[–.12, .15]	[.10, .36]	[.74, .91]
	Negative non-interpersonal dependent events as a stressor measure			
	Aggressive behaviors T2	Negative non-interpersonal dependent events T2	Rumination total T2	Depressive symptoms T2
Aggressive behaviors T1	.74 ***	.00	–.03	–.02
	[.67, .80]	[–.10, .11]	[–.13, .07]	[–.09, .06]
Negative non-interpersonal dependent events T1	.05	.65 ***	.01	.15 **
	[–.07, .16]	[.54, .76]	[–.11, .13]	[.05, .24]
Rumination total T1	–.07	–.07	.54 ***	–.08
	[–.20, .06]	[–.21, .08]	[.42, .67]	[–.19, .02]
Depressive symptoms T1	.07	.12	.24 ***	.83 ***
	[–.06, .19]	[–.02, .26]	[.11, .37]	[.74, .91]
	Negative independent events as a stressor measure			
	Aggressive behaviors T2	Negative independent events T2	Rumination total T2	Depressive symptoms T2
Aggressive behaviors T1	.73 ***	.03	–.04	–.02
	[.66, .80]	[–.08, .15]	[–.14, .06]	[–.10, .06]
Negative independent events T1	.06	.62 ***	.04	.13 **
	[–.04, .17]	[.52, .73]	[–.07, .15]	[.04, .21]
Rumination total T1	–.07	–.11	.53 ***	–.08
	[–.20, .05]	[–.26, .04]	[.41, .66]	[–.18, .03]
Depressive symptoms T1	.07	.14	.24 ***	.85 ***
	[–.05, .19]	[–.00, .28]	[.11, .36]	[.77, .93]

T1 means variable measured at Time 1, and T2 means variable measured at Time 2. Numbers in brackets indicate 95% confidence intervals. * *p* < .05, ** *p* < .01, *** *p* < .001

Supplementary analyses were conducted with brooding or reflection subscales as a rumination measure instead of total RRS scores. The results were largely the same as those in the models using total RRS scores, except for a nonsignificant association between depressive symptoms at Time 1 and reflection at Time 2 (see Tables S1 and S2).

## Discussion

Consistent with Hypothesis 1, aggressive behaviors at baseline were related to an increase in negative interpersonal dependent events, but not negative non-interpersonal dependent events or negative independent events, assessed 8 or 9 weeks later. In addition, the longitudinal association of aggressive behaviors with subsequent negative interpersonal dependent events was significant even after controlling for baseline depressive symptoms and rumination. Students who frequently engage in aggressive behaviors are likely to be rejected by their counterparts and result in interpersonal stress generation because aggressive behaviors physically or psychologically harm others.

The present result of a significant longitudinal association between baseline aggressive behaviors and future increase in negative interpersonal dependent events supports several findings (Molz et al., 2013; Stroud et al., 2015), but extend these by including university students in Japan who were not selected based on diagnostic criteria. Cultural psychology research has indicated that Asians are more interdependent within their significant social units than Westerns (Kitayama et al., 2009; Markus & Kitayama, 1991; Park et al., 2016). Such differences in self-construal between Asians and Westerns could affect the relationship between aggressive behaviors and negative interpersonal dependent events. Aggressive behaviors might be more detrimental to interpersonal relationships in Japanese than in Westerners because Japanese tend to emphasize harmonious interpersonal relationships more strongly, although other possibilities are also feasible. This study only investigated a sample of university students in Japan. Therefore, the study could not compare the magnitude of relationships between baseline aggressive behaviors and future experiences of negative interpersonal dependent events between Japanese and Western populations. Future studies should include Japanese and Western university student participants and use identical measures to examine possible cultural differences in the effects of aggressive behaviors on interpersonal stress generation.

Results showed that all categories of negative events were associated with an increase in subsequent depressive symptoms. These results support Hypothesis 2 and concur with those of many previous studies in which the experience of negative events was found to increase the risk of developing depressive symptoms and depressive disorders (for reviews, see Hammen, 2005; Monroe et al., 2014). The influences of negative interpersonal dependent events, negative non-interpersonal dependent events, and negative independent events on depressive symptoms were very similar (see Table 3). A previous study (Hasegawa et al., 2021b) reported concurrent correlations with depressive symptoms that were significantly stronger for negative dependent events than for negative independent events, and in the present study, concurrent associations between each category of negative events and depressive symptoms were in the same direction (see Table 2). The present findings indicate that the differences in the magnitude of the concurrent correlations with depressive symptoms cannot be explained by different influences of the categories of negative events on depressive symptoms.

The present study also found that baseline depressive symptoms were not significantly associated with any category of negative events experienced in the follow-up period.^4^ Therefore, concurrent associations with depressive symptoms that were stronger for negative dependent events than for negative independent events could not be explained by effects of depressive symptoms on stress generation. The nonsignificant associations between depressive symptoms and subsequent negative dependent events found here are inconsistent with the vast majority of previous results on stress generation effects of depression (Liu & Alloy, 2010). This inconsistency may be related to the fact that this study was conducted under the unusual situation of the global COVID-19 pandemic.

In this study, none of the categories of negative event at baseline were significantly associated with subsequent rumination, and baseline rumination was not significantly associated with subsequent depressive symptoms.^5^ These results do not support Hypotheses 3 and 4. Consistent with the goal progress theory of rumination (Martin et al., 2004) and the psychobiological theory of depression (Slavich et al., 2010), previous studies have shown that frequent exposure to negative events is associated with an increase in rumination (Michl et al., 2013; Hamilton et al., 2015). In these studies, negative event experiences assessed with self-report measures had a smaller effect on rumination (Michl et al., 2013) than those assessed with a structured interview (Hamilton et al., 2015). It is possible that assessment with a self-report measure of negative events may have weakened the influence of negative event experiences on rumination in the present study, because self-reports of negative event experiences can be affected by participant characteristics, such as interpretation bias (Liu, 2013).

The nonsignificant association between rumination and subsequent depressive symptoms was not consistent with many previous longitudinal studies (Nolen-Hoeksema et al., 2008). The predictive power of scores on the Japanese RRS on future depressive symptoms may be relatively weaker than that of the original RRS. For example, the total score on the Japanese RRS predicted increased depressive symptoms assessed 4 and 8 weeks later (Hasegawa et al., 2013, 2018), but not 6 months later (Hasegawa et al., 2015). The Japanese RRS has been used less often than the original RRS to examine the longitudinal relationship between rumination and subsequent depressive symptoms. More effort is necessary to examine the relationship of Japanese RRS scores with future depressive symptoms.

In summary, the present study suggests that aggressive behaviors are a factor leading to interpersonal stress generation. In addition, negative interpersonal dependent events, negative non-interpersonal dependent events, and negative independent events were associated with an increase in depressive symptoms 8 or 9 weeks later, and all negative events had similar predictive powers for depressive symptoms. This study adopted a longitudinal design to examine causality between variables by using a specific measure of aggressive behaviors and including university student participants in Japan that were not selected based on diagnostic criteria, and thus extended previous examinations of the causes and consequences of stress generation conducted in the US.

Several limitations of the present study should be noted in addition to the issue discussed above. The sample consisted only of university students, making it unclear whether the results are generalizable to other age groups or to clinical samples. In addition, the sample size was inadequate for a multiple group path analysis to compare the associations between each variable for male and female groups. Future studies are needed to replicate these findings with different and larger samples. Furthermore, all variables were assessed with self-report measures. More sophisticated assessment tools, such as structured clinical interviews, should be used in future work. Finally, the study was conducted under the unusual situation of the COVID-19 pandemic in Japan. These circumstances may have affected the present findings, although the results obtained in such a situation may have important implications. A future investigation after the pandemic should replicate the present research.

## Supplementary Information

ESM 1 (DOCX 44.7 kb)

## Data Availability

This study’s dataset can be found at the Open Science Framework [https://osf.io/83prm/].

## Footnotes

1 At Hirosaki University, all classes were conducted online for three weeks in the follow-up period due to the spread of the COVID-19, whereas all classes were held face-to-face during the rest of the follow-up period. At Senshu University, only seminars and practical training were conducted face-to-face, and the other classes were held online throughout the study period. At Tokai Gakuin University, all classes were held face-to-face during the period when the final sample answered the questionnaires. At Tokushima University, all classes were held face-to-face for approximately the first two weeks of the follow-up period, and after that, all classes other than seminars and practical training were conducted online.

2 Liu and Alloy (2010) indicated that the shortest time interval in previous longitudinal studies not assessing negative events daily was five weeks. This study set a more extended follow-up period than the shortest period used in previous studies.

3 The sample size of the data on which paired *t*-tests were performed was slightly different from those shown in Table 1 because a few participants in the final sample did not complete specific scales at both time points.

4 In order to examine whether the nonsignificant association between depressive symptoms and stress generation was due to the influence of other study variables, we conducted supplementary path analyses using only depressive symptoms and each category of negative events at both time points. Baseline depressive symptoms were not significantly associated with subsequent negative interpersonal dependent events (*β* = .01, *p* = .846), negative non-interpersonal dependent events (*β* = .09, *p* = .147), or negative independent events (*β* = .08, *p* = .190). These results suggest that the nonsignificant associations between depressive symptoms and stress generation could not be explained by effects of baseline aggressive behaviors and rumination.

5 Path analyses using only each category of negative events and rumination at both time points showed that negative interpersonal dependent events, negative non-interpersonal dependent events, and negative independent events at baseline were not significantly associated with subsequent rumination (*β*s ranged from .05 to .08, *p*s ≥ .147). In addition, path analysis using only rumination and depressive symptoms at both time points showed that baseline rumination was not significantly associated with subsequent depressive symptoms (*β* = –.03, *p* = .541). These results suggest that the nonsignificant associations between these variables are not explained by covariates.
