# Supplementary material for: Causes and consequences of stress generation: Longitudinal associations of negative events, aggressive behaviors, rumination, and depressive symptoms
Source: Curr Psychol. 2022 Feb 23:1–10. Online ahead of print. doi: 10.1007/s12144-022-02859-9 (PMC8864461; doi:10.1007/s12144-022-02859-9)
Supplement: Supplementary file 1 — (DOCX 44.7 kb) [file 12144_2022_2859_MOESM1_ESM.docx]

**Table S1**

*Standardized estimates in each model of path analysis using brooding subscale as a rumination measure*

|  | Negative interpersonal dependent events as a stressor measure | | | | | | | |
| --- | --- | --- | --- | --- | --- | --- | --- | --- |
| Independent variables | Aggressive behaviors  T2 | | Negative interpersonal  dependent events T2 | | Brooding T2 | | Depressive symptoms T2 | |
| Aggressive behaviors T1 | .73 | *** | .12 | * | –.02 |  | –.03 |  |
|  | [.66, .80] | | [.01, .22] | | [–.13, .08] | | [–.11, .05] | |
| Negative interpersonal  dependent events T1 | .05 |  | .67 | *** | .08 |  | .14 | ** |
|  | [–.07, .16] | | [.57, .77] | | [–.05, .21] | | [.05, .23] | |
| Brooding T1 | –.09 |  | –.05 |  | .43 | *** | –.06 |  |
|  | [–.21, .03] | | [–.18, .08] | | [.31, .56] | | [–.16, .03] | |
| Depressive symptoms T1 | .08 |  | .04 |  | .26 | *** | .82 | *** |
|  | [–.04, .20] | | [–.09, .16] | | [.12, .39] | | [.74, .89] | |
|  |  |  |  |  |  |  |  |  |
|  | Negative non-interpersonal dependent events as a stressor measure | | | | | | | |
|  | Aggressive behaviors  T2 | | Negative non-interpersonal  dependent events T2 | | Brooding T2 | | Depressive symptoms T2 | |
| Aggressive behaviors T1 | .73 | *** | –.00 |  | –.01 |  | –.02 |  |
|  | [.67, .80] | | [–.11, .10] | | [–.12, .10] | | [–.10, .06] | |
| Negative non-interpersonal  dependent events T1 | .06 |  | .65 | *** | .01 |  | .14 | ** |
|  | [–.06, .17] | | [.54, .76] | | [–.12, .14] | | [.05, .23] | |
| Brooding T1 | –.10 |  | –.06 |  | .45 | *** | –.07 |  |
|  | [–.22, .02] | | [–.20, .07] | | [.32, .58] | | [–.17, .03] | |
| Depressive symptoms T1 | .08 |  | .12 |  | .27 | *** | .82 | *** |
|  | [–.04, .20] | | [–.02, .25] | | [.14, .41] | | [.74, .90] | |
|  |  |  |  |  |  |  |  |  |
|  | Negative independent events as a stressor measure | | | | | | | |
|  | Aggressive behaviors  T2 | | Negative independent events T2 | | Brooding T2 | | Depressive symptoms T2 | |
| Aggressive behaviors T1 | .73 | *** | .03 |  | –.01 |  | –.02 |  |
|  | [.66, .80] | | [–.08, .14] | | [–.12, .10] | | [–.10, .05] | |
| Negative independent  events T1 | .07 |  | .61 | *** | .03 |  | .12 | ** |
|  | [–.04, .18] | | [.51, .72] | | [–.09, .15] | | [.04, .21] | |
| Brooding T1 | –.10 |  | –.08 |  | .45 | *** | –.06 |  |
|  | [–.22, .02] | | [–.22, .06] | | [.32, .58] | | [–.16, .04] | |
| Depressive symptoms T1 | .08 |  | .12 |  | .27 | *** | .84 | *** |
|  | [–.03, .20] | | [–.02, .26] | | [.14, .40] | | [.76, .91] | |
| *Note*. T1 means variable measured at Time 1, and T2 means variable measured at Time 2. Numbers in brackets indicate 95% confidence intervals. * *p* < .05, ** *p* < .01, *** *p* < .001. | | | | | | | | |

**Table S2**

*Standardized estimates in each model of path analysis using reflection subscale as a rumination measure*

|  | Negative interpersonal dependent events as a stressor measure | | | | | | | |
| --- | --- | --- | --- | --- | --- | --- | --- | --- |
| Independent variables | Aggressive behaviors  T2 | | Negative interpersonal  dependent events T2 | | Reflection T2 | | Depressive symptoms T2 | |
| Aggressive behaviors T1 | .74 | *** | .11 | * | –.04 |  | –.03 |  |
|  | [.67, .81] | | [.01, .21] | | [–.15, .07] | | [–.11, .05] | |
| Negative interpersonal  dependent events T1 | .04 |  | .65 | *** | .02 |  | .13 | ** |
|  | [–.08, .15] | | [.54, .75] | | [–.11, .14] | | [.04, .22] | |
| Reflection T1 | –.04 |  | .05 |  | .65 | *** | –.01 |  |
|  | [–.15, .06] | | [–.06, .16] | | [.55, .75] | | [–.10, .07] | |
| Depressive symptoms T1 | .05 |  | .00 |  | .09 |  | .79 | *** |
|  | [–.06, .15] | | [–.11, .11] | | [–.03, .20] | | [.72, .85] | |
|  |  |  |  |  |  |  |  |  |
|  | Negative non-interpersonal dependent events as a stressor measure | | | | | | | |
|  | Aggressive behaviors  T2 | | Negative non-interpersonal  dependent events T2 | | Reflection T2 | | Depressive symptoms T2 | |
| Aggressive behaviors T1 | .74 | *** | –.01 |  | –.03 |  | –.02 |  |
|  | [.67, .81] | | [–.11, .10] | | [–.14, .07] | | [–.10, .06] | |
| Negative non-interpersonal  dependent events T1 | .04 |  | .63 | *** | –.02 |  | .12 | ** |
|  | [–.07, .15] | | [.52, .73] | | [–.14, .10] | | [.03,.22 ] | |
| Reflection T1 | –.04 |  | .03 |  | .66 | *** | –.01 |  |
|  | [–.15, .06] | | [–.09, .14] | | [.57, .75] | | [–.09, .08] | |
| Depressive symptoms T1 | .04 |  | .08 |  | .10 |  | .79 | *** |
|  | [–.07, .15] | | [–.04, .20] | | [–.02, .22] | | [.72, .85] | |
|  |  |  |  |  |  |  |  |  |
|  | Negative independent events as a stressor measure | | | | | | | |
|  | Aggressive behaviors  T2 | | Negative independent events T2 | | Reflection T2 | | Depressive symptoms T2 | |
| Aggressive behaviors T1 | .73 | *** | .03 |  | –.04 |  | –.02 |  |
|  | [.66, .80] | | [–.08, .15] | | [–.15, .07] | | [–.10, .05] | |
| Negative independent  events T1 | .06 |  | .61 | *** | .01 |  | .11 | * |
|  | [–.05, .17] | | [.50, .71] | | [–.10, .13] | | [.02, .20] | |
| Reflection T1 | –.05 |  | –.04 |  | .65 | *** | –.00 |  |
|  | [–.15, .06] | | [–.16, .08] | | [.56, .75] | | [–.09, .08] | |
| Depressive symptoms T1 | .04 |  | .09 |  | .09 |  | .81 | *** |
|  | [–.06, .15] | | [–.03, .21] | | [–.03, .20] | | [.75, .87] | |
| Note: T1 means variable measured at Time 1, and T2 means variable measured at Time 2. Numbers in parentheses indicate 95% confidence intervals. * *p* < .05, ** *p* < .01, *** *p* < .001. | | | | | | | | |
